# Associations between Amygdala-Prefrontal Functional Connectivity and Age Depend on Neighborhood Socioeconomic Status

**DOI:** 10.1093/texcom/tgaa033

**Published:** 2020-07-23

**Authors:** Bruce Ramphal, Mariah DeSerisy, David Pagliaccio, Elizabeth Raffanello, Virginia Rauh, Gregory Tau, Jonathan Posner, Rachel Marsh, Amy E Margolis

**Affiliations:** New York State Psychiatric Institute and Department of Psychiatry, Vagelos College of Physicians and Surgeons, Columbia University, New York, NY 10032, USA; Department of Psychology, Fordham University, Bronx, NY 10458, USA; New York State Psychiatric Institute and Department of Psychiatry, Vagelos College of Physicians and Surgeons, Columbia University, New York, NY 10032, USA; New York State Psychiatric Institute and Department of Psychiatry, Vagelos College of Physicians and Surgeons, Columbia University, New York, NY 10032, USA; Department of Population and Family Health, Mailman School of Public Health, Columbia University, New York, NY 10032, USA; New York State Psychiatric Institute and Department of Psychiatry, Vagelos College of Physicians and Surgeons, Columbia University, New York, NY 10032, USA; New York State Psychiatric Institute and Department of Psychiatry, Vagelos College of Physicians and Surgeons, Columbia University, New York, NY 10032, USA; New York State Psychiatric Institute and Department of Psychiatry, Vagelos College of Physicians and Surgeons, Columbia University, New York, NY 10032, USA; New York State Psychiatric Institute and Department of Psychiatry, Vagelos College of Physicians and Surgeons, Columbia University, New York, NY 10032, USA

**Keywords:** anxiety, brain development, fMRI, functional connectivity, stress acceleration

## Abstract

Although severe early life stress has been shown to accelerate the development of frontolimbic resting-state functional connectivity (RSFC), less is known about the effects of socioeconomic disadvantage, a prolonged and multifaceted stressor. In a cross-sectional study of 127 participants aged 5–25, we examined whether lower neighborhood socioeconomic status (SES; measured by Area Deprivation Index and neighborhood poverty and educational attainment) was associated with prematurely reduced amygdala-ventromedial prefrontal cortex (vmPFC) RSFC. We further tested whether neighborhood SES was more predictive than household SES and whether SES effects on connectivity were associated with anxiety symptoms. We found reduced basolateral amygdala-vmPFC RSFC at earlier ages in participants from more disadvantaged neighborhoods; this effect was unique to neighborhood SES and absent for household SES. Furthermore, this reduced connectivity in more disadvantaged youth and increased connectivity in more advantaged youth were associated with less anxiety; children who deviated from the connectivity pattern associated with their neighborhood SES had more anxiety. These results demonstrate that neighborhood socioeconomic disadvantage is associated with accelerated maturation of amygdala-vmPFC RSFC and suggest that the pathophysiology of pediatric anxiety depends on a child’s neighborhood socioeconomic characteristics. Our findings also underscore the importance of examining SES effects in studies of brain development.

## Introduction

Socioeconomic status (SES) indexes social prestige and financial resources and has profound and lasting mental health effects (Bradley and Corwyn 2002b; Hackman and Farah 2009; Reiss 2013). At the household level, SES is commonly assessed by measures such as occupation, educational attainment, and household income. Importantly, household SES often covaries with factors linked with mental wellbeing, such as access to stable and secure shelter (Gilman et al. 2003), mental healthcare access (Hodgkinson et al. 2017), household stability (Evans et al. 2005), and exposure to stressful childhood experiences (Goldmann et al. 2011). These stressors likely contribute to critical socioeconomic disparities in health outcomes (Baum et al. 1999). At the neighborhood level, SES is often assessed as both the combined SES of a community’s constituent households as well as shared aspects of a community’s material and social infrastructure, including health care resources, healthy food availability, neurotoxic environmental burden, exposure to local violence, and social cohesion (Macintyre et al. 2002; Wilson et al. 2004; Braveman et al. 2005; Xue et al. 2005; Kohen et al. 2008; Meyer et al. 2014; Burt et al. 2016; Chang et al. 2016; DeVylder et al. 2018). Lower household and neighborhood SES are linked to poorer mental and physical health outcomes, including greater risk of anxiety, depression, and substance abuse (Leventhal and Brooks-Gunn 2000; Boardman et al. 2001; Singh et al. 2002; Singh 2003; Cutrona et al. 2006; Menec et al. 2010; Slopen et al. 2010; Spijkers et al. 2012; Ruscio et al. 2017).

Some work points to dissociable effects of household and neighborhood SES. Specifically, observational and experimental data suggest that, relative to household SES, neighborhood SES better explains variance in externalizing behaviors (Brooks-Gunn et al. 1993; Chase-Lansdale and Gordon 1996; Ludwig et al. 2001; Jackson et al. 2009) and internalizing symptoms (Chase-Lansdale and Gordon 1996; Leventhal and Brooks-Gunn 2003; Leventhal and Dupere 2011), particularly in children. The relatively strong effects of neighborhood disadvantage on child development may in fact be mediated by lack of neighborhood cohesion and engagement in peer-encouraged antisocial behavior (Ludwig et al. 2001; Kohen et al. 2008). Despite the known association between household and neighborhood SES on mental health and the dissociable effects described above, few neuroimaging studies have investigated the dissociable neural consequences of household and neighborhood SES (Gianaros et al. 2017; Miller et al. 2018) or how these effects underlie risk for psychopathology (Marshall et al. 2018; Tomlinson et al. 2020).

The human neuroscience of socioeconomic status is an emerging field that builds on a robust literature on early life stress in animals and humans (Hackman et al. 2010; Farah 2018). Importantly, SES and stress are not identical; nevertheless, low SES families have greater perceived (Stronks et al. 1998) and physiological markers of stress (Seeman et al. 2010; Hackman et al. 2012; Schulz et al. 2012; Vliegenthart et al. 2016; Finegood et al. 2017; Theall et al. 2017; Roubinov et al. 2018), and stress has been understood as a primary mechanism by which socioeconomic disadvantage confers its deleterious effects (Baum et al. 1999; Evans and Schamberg 2009; Hackman et al. 2010; Merz et al. 2019). In rodents, early life stress triggers developmental cascades characterized by hypothalamus-pituitary-adrenal (HPA) axis dysregulation (Maniam et al. 2014), altered neural development in psychiatrically important brain regions such as the ventromedial prefrontal cortex, amygdala, hippocampus, and their connections (McEwen 2013; van Bodegom et al. 2017), and aberrant behavioral development (Bolton et al. 2017). Indeed, human studies of severe early life stress as well as studies of household socioeconomic disadvantage have converged in implicating these very same neuroendocrine pathways in the etiology of stress-related psychopathology with disadvantaged individuals exhibiting altered stress physiology (Lupien et al. 2000; Cohen et al. 2006; Raymond et al. 2018), reduced hippocampal volumes (Hanson et al. 2015; Barch et al. 2016; Merz et al. 2019), and increased internalizing (Everson et al. 2002; Heim and Binder 2012; Gee, Gabard-Durnam, et al. 2013a; Ruscio et al. 2017) and externalizing (Kim et al. 2003; Russell et al. 2016) psychopathology.

One consistent finding in rodent and human research of early life stress, but not yet extended to human SES research, is that disadvantage precipitates precocious development of the amygdala-ventromedial prefrontal cortex emotion regulation pathway (more generally termed the “stress acceleration hypothesis”) (Ono et al. 2008; Callaghan and Richardson 2011; Gee, Gabard-Durnam, et al. 2013a; Ishikawa et al. 2015; Callaghan and Tottenham 2016; Thijssen et al. 2017; VanTieghem and Tottenham 2018; Tottenham 2020). Whereas functional connectivity between the amygdala and the ventromedial prefrontal cortex normally decreases (shifts from positive to negative or near zero) over the course of child development to promote emotion regulation (Lee et al. 2012; Gee, Humphreys, et al. 2013b; Motzkin et al. 2015; Jalbrzikowski et al. 2017), this shift is accelerated in individuals exposed to high levels of early life stress (Gee, Gabard-Durnam, et al. 2013a; Wolf and Herringa 2016). Interestingly, although stress itself is associated with increased risk for internalizing psychopathology (Hicks et al. 2009; McLaughlin et al. 2010; Carr et al. 2013), precociously negative amygdala-vmPFC development seems to confer resilience against symptoms, and positive connectivity is associated with more symptoms in stressed individuals (Gee, Gabard-Durnam, et al. 2013a; Wolf and Herringa 2016). In contrast, other studies that do not directly investigate stress effects have found reduced connectivity in children with anxiety (Hamm et al. 2014). One possible explanation for these divergent findings is that the behavioral relevance of amygdala-vmPFC functional connectivity may be context-dependent, such that reduced connectivity is protective in some pediatric contexts but not in others (Tottenham 2020).

It remains unknown whether socioeconomic disadvantage has a similar effect to early life stress on associations between age and amygdala-vmPFC resting-state functional connectivity (RSFC), whether household and neighborhood SES have convergent or distinct effects on connectivity and to what extent effects on connectivity are associated with anxiety symptoms. Here, we sought to investigate whether the stress acceleration hypothesis extends to associations between amygdala-vmPFC resting-state functional connectivity and neighborhood or household SES. Specifically, we hypothesized that participants from high SES neighborhoods would demonstrate a normative pattern of amygdala-vmPFC connectivity, such that RSFC would vary inversely with age. In contrast, we expected that participants from lower SES neighborhoods would demonstrate more adult-like connectivity at younger ages relative to higher SES participants, consistent with findings from individuals with early life stress (Gee, Gabard-Durnam, et al. 2013a). Second, given findings suggesting distinct contributions of neighborhood and household SES to mental health (Brooks-Gunn et al. 1993; Chase-Lansdale and Gordon 1996; Ludwig et al. 2001; Leventhal and Brooks-Gunn 2003; Jackson et al. 2009), we expected neighborhood effects on connectivity to be dissociable from household effects. Third, consistent with prior findings in individuals with severe early life stress (Gee, Gabard-Durnam, et al. 2013a), we hypothesized that more adult-like connectivity in low neighborhood SES youth would be associated with fewer anxiety symptoms.

## Materials and Methods

### Participants

Data were pooled for secondary data analysis from 151 participants ranging from 5 to 25 years old who were all recruited from the New York City area for several case-control studies between 2011 and 2017 as healthy controls (*n* = 84) or patients with attention deficit hyperactivity disorder (ADHD; *n* = 67; no other clinical groups were included; see Supplementary Table 1 for recruitment strategies) (Marsh et al. 2011; Tau et al. 2014; Cha et al. 2015; Davis et al. 2018). Healthy participants exhibited no psychiatric diagnosis as determined by diagnostic interview (Kiddie-Schedule for Affective Disorders, Diagnostic Interview Schedule for Children, or Structured Clinical Interview for DSM-IV). Participants with ADHD were included to maximize sample size. Furthermore, participants with ADHD were not expected to differ in amygdala-vmPFC connectivity (Posner et al. 2011; Posner et al. 2013; Castellanos and Aoki 2016). Statistical methods were employed to test whether ADHD diagnosis accounted for any observed effects (see Statistical Analyses). The Institutional Review Board of the New York State Psychiatric Institute approved this study.

### Socioeconomic Status

Neighborhood SES was measured using the Area Deprivation Index (ADI) (https://www.neighborhoodatlas.medicine.wisc.edu/) (Singh 2003; Kind et al. 2014; Kind and Buckingham 2018). The ADI is a widely used measure of neighborhood SES derived on the census block level based on a factor analysis of neighborhood characteristics from the 2013 American Community Survey 5-Year Estimates, including poverty prevalence, adult educational attainment, home value, and household crowding (Marshall et al. 2020). Census block groups correspond to areas with populations approximately ranging from 600 to 3000 (census.gov.programs-surveys/geography/about/glossary.html). The ADI reports neighborhood SES as national percentiles with the highest percentiles corresponding to the most disadvantaged neighborhoods in the United States. The ADI was calculated based on participant address at the time of scanning; participants located in crowded neighborhoods (i.e., college campus) were excluded because the ADI is not calculated for those communities.

According to the factor analysis used to develop the ADI, the score is most heavily driven by the proportion of individuals in the census block group with incomes below 150% of the poverty threshold and the proportion of individuals 25 years or older with at least a high school diploma contribute (Kind et al. 2014). We acquired these values for each participant’s neighborhood from the American Community Survey (https://data.census.gov/cedsci/). We then determined whether neighborhood poverty or neighborhood educational characteristics contributed more to any observed neighborhood effects.

**Figure 1:**
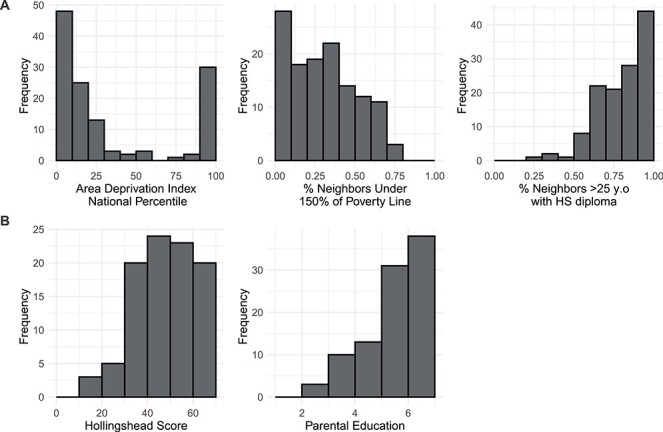
Distributions of (*A*) neighborhood and (*B*) household socioeconomic measures. Higher Area Deprivation Index National Percentile corresponded to lower neighborhood SES. Hollingshead scores range from 8 to 66 with lower numbers corresponding to lower household SES. Parental education is an ordinal variable ranging from 1 to 7: 1 = less than middle school; 2 = completed middle school; 3 = some high school; 4 = completed high school/attained GED; 5 = some college/associate’s degree; 6 = bachelor’s degree; 7 = graduate degree.

Given the bimodal distribution of ADI percentiles in the current sample, the variable was analyzed in three groups: low (90–100), middle (11–89), and high neighborhood SES (1–10; Fig. 1). Participants classified as having low neighborhood SES resided in neighborhoods in which, on average, 54.3% of families had incomes under 150% of the poverty line, while this rate was 25.9% and 20.0% for middle and high neighborhood SES families, respectively (Supplementary Table 2). ADI was also analyzed as a continuous variable.

Household socioeconomic status was measured with the Hollingshead Four-Factor Index of Social Status (Hollingshead 1975). The Hollingshead score is determined as a weighted sum of parental education level (1–7) and parental occupation scores (1–9) with higher numbers representing greater educational attainment, occupational prestige, and income (see Supplemental Methods for coding). For households with two employed parents, the score was calculated as the average of both parents’ scores; for households with one parent, the score was calculated for that one parent. The Hollingshead scores derived from this averaging procedure are highly correlated with scores derived from scores derived from maximal parental education and occupation (*r* = 0.92); nevertheless, results using this method are presented in the supplement. We acknowledge that single-parent status is a contributor to SES (McLanahan and Sandefur 1994; Bradley and Corwyn 2002a; Conger et al. 2010); however, because only 10 participants were from single-parent households, we were unable to examine its effects in this study. Parental education was also analyzed independently as an ordinal variable corresponding to the parent with the greatest educational attainment. Household income was not collected in these studies as they had originally intended to examine group differences in brain measures between clinical and control samples. The distributions of all measures of socioeconomic status are presented in Figure 1, and how they vary across studies is presented in Supplementary Figure 1.

### Anxiety

Given the role of amygdala-vmPFC connectivity in anxiety disorders (Gee, Gabard-Durnam, et al. 2013a; Hamm et al. 2014; Wolf and Herringa 2016), we examined anxiety symptoms as measured by the Anxious/Depressed subscale T-score of the Child Behavior Checklist (CBCL) in a subset of participants with available data (*n* = 65) (Achenbach 1983). The CBCL was available for participants 6–17 years old (mean = 10.7 years; standard deviation = 3.5 years).

### MRI Acquisition

All MRI data were acquired on a General Electric Signa 3 Tesla LX scanner (Milwaukee, WI). Specific acquisition parameters from each study are presented in Supplementary Table 1. To account for slight differences in MRI acquisition (flip angle, TR, and run length), a three-group dummy code was included as a covariate in all imaging analyses.

### Preprocessing and Motion Correction

MRI data preprocessing was performed using the CONN Toolbox (Whitfield-Gabrieli and Nieto-Castanon 2012). Steps included functional realignment and unwarping, slice timing correction, scrubbing, and simultaneous segmentation and normalization to the Montreal Neurological Institute (MNI) template. Head motion outliers were identified using the ART tools (>0.5 mm framewise displacement or Z > 3 change in global signal). Frames with head motion outlier were regressed in participant-level models along with anatomical nuisance regressors (aCompCor) from white matter (10 components) and cerebrospinal fluid (10 components) (Behzadi et al. 2007). Participants were included in final analyses if they had more than 5 min of functional data uncontaminated by motion. Functional data were band-pass filtered (0.01–0.1 Hz). Supplementary analyses examined models including global signal regression.

### Statistical Analyses

Regions of interest (ROI) included left and right amygdala subregions (basolateral and centromedial; Juelich Histological Atlas with 50% probability threshold) (Amunts et al. 2005) and the ventromedial prefrontal cortex (Harvard-Oxford atlas). The blood oxygen level-dependent (BOLD) time course of each ROI was calculated as the average of the time courses of its constituent voxels. Resting-state functional connectivity between two ROIs was calculated as the Fisher Z-transformed correlation coefficient of their time courses in CONN.

Functional connectivity between the vmPFC and amygdala subregions (4 subregions; 4 connections total) served as dependent variables in multiple linear regression models with age, neighborhood SES group (3 levels), and their interaction as predictors of interest and sex, mean head motion during MRI acquisition, dummy-coded pulse sequence, and ADHD diagnosis as covariates. The high SES group served as the reference group for these analyses as they were expected to show the previously observed negative association between age and connectivity. The specific models used for analyses are detailed in the Supplementary Methods, as well as sample R code (R version 3.5.3) (Core Team 2015). All reported betas are standardized. All model residuals were tested for normality using the Shapiro-Wilk test.

To address our first hypothesis, we first tested if connectivity was inversely associated with age in the high SES group by inspecting the “age” term in each of our models. Next, we inspected the age×SES interaction terms to determine if SES (low or middle vs. high) moderated the association between age and resting-state functional connectivity. In the absence of interaction effects, the effects of dummy-coded SES groups were then inspected to determine whether mean connectivity varied between groups. All 20 significance tests for these analyses (age, age × middle SES, age × low SES, middle SES, and low SES terms for 4 connections) were two-sided, and multiple comparisons correction was performed using false discovery rate (FDR; “*P*.adjust” function from *stats* package). We also investigated whether neighborhood poverty rates or neighborhood educational attainment (the two variables that contribute most to the ADI score) better explained observed effects. To confirm our main findings, post hoc analyses (detailed in Supplementary Methods and Supplementary Results) examined effects of global signal regression, treating neighborhood SES as a continuous variable, quadratic age effects (Shaw et al. 2008), and whether our results might have been driven by the inclusion of participants with ADHD.

To test our second hypothesis that neighborhood SES and household SES would have dissociable effects on the association between age and amygdala-vmPFC RSFC, we included interactions between age and both neighborhood SES and household SES (parental education or Hollingshead score) terms in the same model. We also performed this analysis with only household SES and its interaction with age in the model.

To test our third hypothesis that adult-like connectivity in low rather than high neighborhood SES youth would be associated with reduced anxiety symptoms in the subset of participants with available data, we tested the three-way interaction between age, neighborhood SES (continuous given reduced sample size and power), and amygdala-vmPFC RSFC on anxiety/depression symptoms, controlling for age, sex, motion, and pulse sequence. We note that these results are preliminary given reduced sample size and power.

## Results

### Participants

Of the 151 participants with neuroimaging data and home addresses available, 15 were excluded for having less than 5 min of useable data after motion correction, leaving 136 with useable MRI data. Because 9 participants reported a college campus as their residence, 127 participants had useable imaging data and addresses that could be converted into an ADI score. Sample characteristics are presented in Table 1. Briefly, 127 participants, 5–25 years old, were included in main analyses; 73 were recruited as healthy controls and 54 had ADHD. Forty-eight participants were from high SES neighborhoods, 48 were from middle, and 31 were from low SES neighborhoods. Additional details about neighborhood SES and details about our behavioral subsample are presented in Supplementary Results.

**Table 1:** Sample characteristics

*N* = 127	Mean (SD)/*N* (%)	Range
Sex (female)	61 (48.0%)	Female/male
Age (years)	14.7 (5.3)	5–25
Low neighborhood SES	15.0 (5.9)	6–25
Middle neighborhood SES	14.9 (5.0)	5–23
High neighborhood SES	13.8 (4.9)	6–24
ADHD diagnosis	54 (42.5%)	ADHD/healthy
Neighborhood disadvantage		
Area deprivation index (ADI)	35.4 (38.2)	1–100
Low SES (ADI: 90–100)	31 (24.4%)	
Middle SES (ADI: 11–89)	48 (37.8%)	
High SES (ADI: 1–10)	48 (37.8%)	
Ethnicity		
White	44 (34.6%)	
Hispanic	35 (27.6%)	
Black	25 (19.7%)	
Asian	11 (8.7%)	
Multiracial	4 (3.1%)	
Other/unknown	8 (6.3%)	
Parental education		
Some middle school	0 (0%)	
Completed middle school	0 (0%)	
Some high school	3 (2.4%)	
High school/GED	12 (9.4%)	
Some college/associates	15 (11.8%)	
Bachelor’s degree	32 (25.2%)	
Graduate degree	40 (31.5%)	
Hollingshead	47.5 (12.5)	14–66
Mean head motion (mm)	0.19 (0.17)	0.05–1.15
% Frames useable	90.3 (0.09)	58.2–100

**Figure 2:**
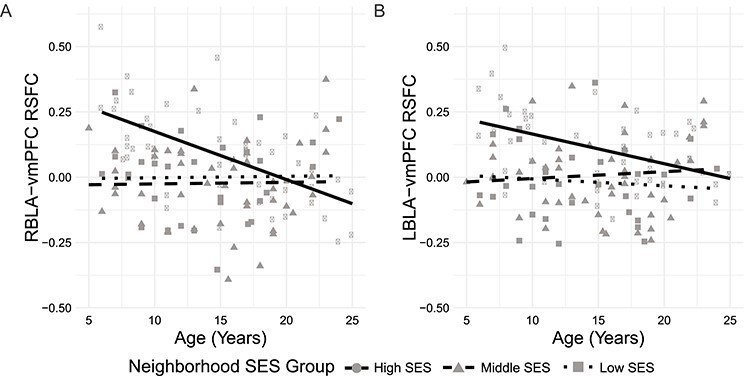
The association between age and basolateral right amygdala (RBLA)-ventromedial prefrontal cortex (vmPFC) resting-state functional connectivity (RSFC) depends on neighborhood socioeconomic status (SES). RBLA-vmPFC connectivity shifts from positive to negative with increasing age among participants with high neighborhood SES. LBLA-vmPFC connectivity is near zero among participants with lower neighborhood SES. Fit lines represent slopes within each neighborhood SES group.

**Table 2:** Effects of age, neighborhood SES, and their interaction on amygdala-vmPFC connectivity

*N* = 127	Age (in high neighborhood SES participants)		Age × neighborhood SES				Neighborhood SES			
			Middle SES > High SES		Low SES > High SES		Middle SES > High SES		Low SES > High SES	
	*β*	*P*	*β*	*P*	*β*	*P*	*β*	*P*	*β*	*P*
RBLA	−0.56	0.00009^*^	0.61	0.002^*^	0.58	0.01^*^	−0.65	0.001^*^	−0.52	0.02
LBLA	−0.24	0.092	0.43	0.028	0.20	0.37	−0.56	0.005^*^	−0.76	0.0009^*^
RCMA	−0.32	0.037	0.26	.22	0.35	0.16	−0.42	0.05	−0.32	0.18
LCMA	−0.19	0.21	0.06	.78	0.39	0.11	−0.28	0.18	−0.31	0.19

*Notes:*

1. In all analyses, the high socioeconomic status (SES) group served as the reference group, meaning that the age effect beta refers to the effect of age within the high SES group, moderation betas refer to the difference between age effects in middle or low SES groups and the high SES group, and main SES effects betas refer to group differences in covariate-adjusted mean connectivity; R/LBLA = right/left basolateral amygdala; R/LCMA = right/left centromedial amygdala

2. All models control for sex, head motion, pulse sequence, and ADHD diagnosis; reported betas are standardized; ^*^P < 0.05 with FDR-correction across all 20 regression coefficient significance tests

### Neighborhood SES and Amygdala-vmPFC Functional Connectivity

The neighborhood SES × age interaction predicted resting-state functional connectivity (RSFC) between right basolateral amygdala (RBLA) and ventromedial prefrontal cortex (vmPFC). Among participants from high but not low or middle SES neighborhoods, age was inversely associated with connectivity, such that connectivity shifted from positive in younger participants to negative connectivity in older participants (Fig. 2*A*; Table 2). The proportion of young participants (age < mean age of 14.7 years) with positive connectivity varied with neighborhood SES such that 90%, 53%, and 63% of young participants had positive connectivity in the high, middle, and low neighborhood SES groups, respectively (*χ*^2^ = 7.07, *P* = 0.029).

The interaction between age and neighborhood SES did not predict left BLA-vmPFC RSFC. However, compared with high SES individuals, individuals from middle and low SES neighborhoods had significantly lower connectivity between left BLA and vmPFC, such that mean connectivity was 0.11, 0.01, and −0.02 in the high, middle, and low SES groups, respectively (Fig. 2*B*; Table 2). The proportion of participants with positive connectivity varied with neighborhood SES such that 73%, 59%, and 36% of participants had positive connectivity in the high, middle, and low neighborhood SES groups (*χ*^2^ = 7.97, *P* = 0.018). No age, SES, or interaction effects survived multiple comparisons correction for connectivity between right or left centromedial amygdala and vmPFC (Table 2). Analyses that included global signal regression or treated neighborhood SES as a continuous variable yielded similar results (Supplementary Tables 3 and 4).

**Figure 3:**
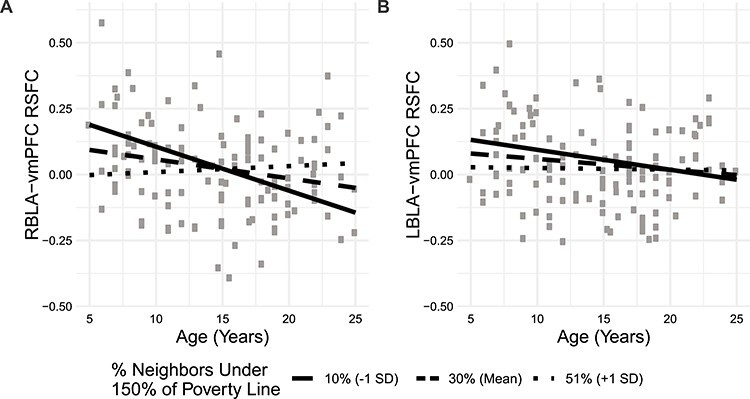
The association between age and right basolateral amygdala (RBLA)-ventromedial prefrontal cortex (vmPFC) resting-state functional connectivity (RSFC) depends on neighborhood poverty rates. RBLA-vmPFC connectivity shifts from positive to negative with increasing age among participants with less neighborhood poverty. Simple slope fit lines are displayed.

The proportion of neighbors below 150% of the poverty threshold interacted with age to predict RBLA-vmPFC functional connectivity (Fig. 3; Table 3); the proportion of neighbors 25 years or older with a high school (HS) diploma also interacted with age to predict connectivity, but did not pass multiple comparisons correction. Post hoc analysis confirmed that age only predicted connectivity among advantaged participants (% neighbors under 150% poverty line <24% and % neighbors 25 years or older with HS diploma >90%; Supplementary Fig. 2). When we included both of these neighborhood socioeconomic measures and their interactions with age in the same model, poverty remained a significant moderator while HS graduation did not [*P*(age × poverty) = 0.029; *P*(age × education) = 0.989]. Neither neighborhood poverty nor educational attainment was significantly associated with LBLA-vmPFC RSFC (Table 3).

We detected a significant interaction between age^2^ and neighborhood SES such that low SES children had a parabolic (in addition to linear) trajectory of RBLA-vmPFC connectivity (Supplementary Table 5).

ADHD had no effect on connectivity in main analyses (*P*s > 0.27). Post hoc analyses yielded neither any significant three-way interactions (SES group × age × ADHD diagnosis; Supplementary Table 6), nor any age × ADHD interactions (Supplementary Table 7), indicating that ADHD diagnosis did not underlie observed interactions between age and neighborhood SES.

### Neighborhood SES, Household SES, and Amygdala-vmPFC Functional Connectivity

Area deprivation index (ADI) was correlated with both parental education and Hollingshead (*r* = −0.37, *P* = 0.0001; *r* = −0.36, *P* = 0.0003, respectively). Neighborhood SES × age and neighborhood SES terms remained significant upon the addition of household SES to our first model; household SES and its interaction with age did not predict RSFC (Table 4). Results were nearly identical when using Hollingshead score as determined by maximal household occupation/education (Supplementary Table 8) and when using 3-group formulations of the Hollingshead and parental education (Supplementary Table 9). We also tested whether neighborhood poverty was a stronger moderator than household SES and found convergent results (Supplementary Table 10). Furthermore, neither household SES nor the household SES × age interaction was associated with RSFC in models without neighborhood SES (Supplementary Table 11). All models reported had normally distributed residuals (Shapiro-Wilk test *P*-values >0.12).

### Symptom Associations

**Figure 4:**
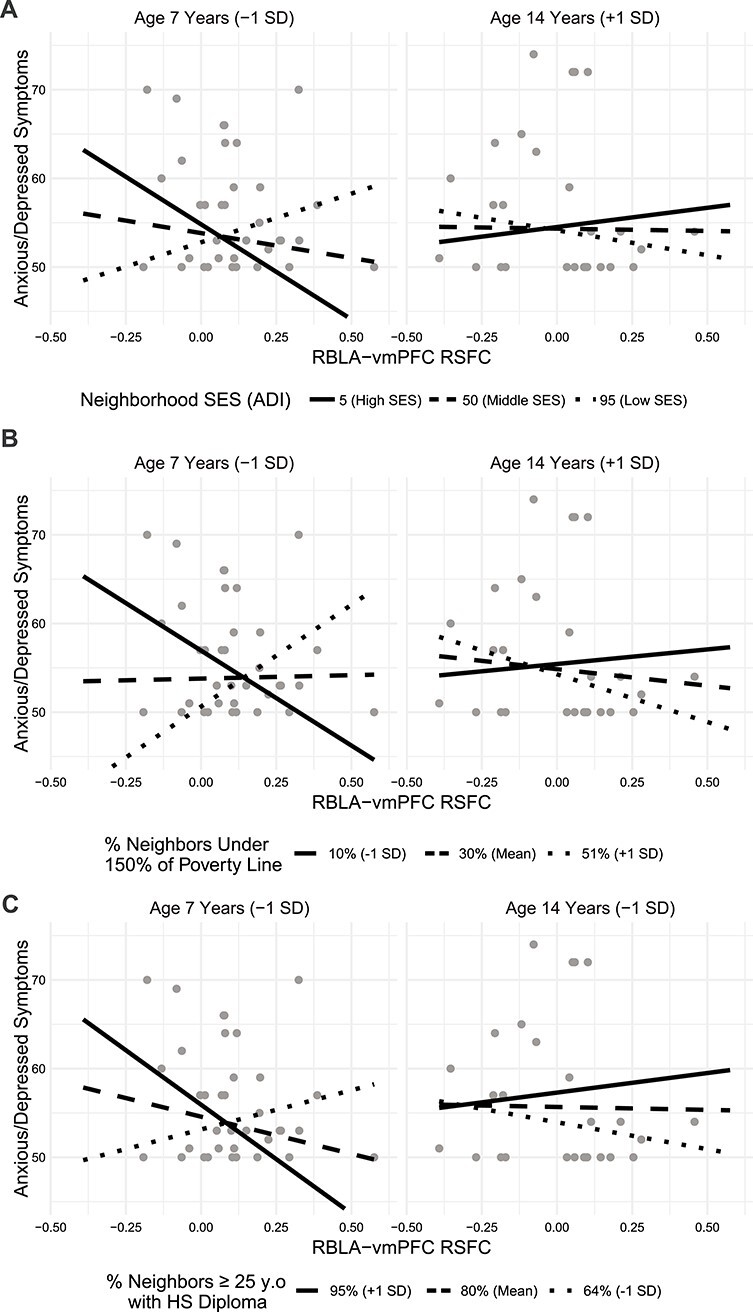
The association between RBLA-vmPFC resting-state connectivity and anxious/depressed symptoms depends on neighborhood socioeconomic status (SES) and age. Younger participants from low SES communities have a positive association between connectivity and anxiety; younger participants from high SES communities have an inverse relationship. Simple slope fit lines are displayed.

A three-way interaction between neighborhood SES, age, and RBLA-vmPFC resting-state connectivity predicted CBCL anxiety symptoms, such that low neighborhood SES children (participants at the lower end of the age range) with characteristically more negative connectivity had fewer symptoms (Table 5, Fig. 4*A*, *P* = 0.006), as did high SES children with characteristically more positive connectivity. Conversely, low SES children with positive connectivity and high SES children with low connectivity had more anxious/depressed symptoms. In adolescents (participants at the higher end of the age range), connectivity did not predict symptoms regardless of SES (Fig. 4). This three-way interaction was also significant when using neighborhood poverty and neighborhood high school completion (Fig. 4*B*,*C*, Table 5), but not when using household SES measures (Supplementary Table 12). No associations were found between symptoms and LBLA-vmPFC functional connectivity (Table 5).

## Discussion

### Summary of Results

Here, we have shown that amygdala subregional-vmPFC RSFC depends on neighborhood socioeconomic status. First, we found that RBLA-vmPFC RSFC was inversely correlated with age in individuals from the most socioeconomically advantaged neighborhoods, while no relationship between age and RSFC was detected in those from less advantaged communities. Further, LBLA-vmPFC RSFC was more negative in participants from less advantaged communities. Importantly, we show this effect using three measures of neighborhood SES and provide evidence that neighborhood economic (rather than educational) factors more precisely explain the observed results. Second, these effects were uniquely explained by neighborhood characteristics and not household socioeconomic measures of education and Hollingshead score. Finally, we provide preliminary evidence that among younger individuals from more disadvantaged communities, negative connectivity may protect against anxiety and depression symptoms, while positive connectivity may protect against these symptoms in younger individuals from more advantaged communities.

### Potential Mechanisms Linking Neighborhood Disadvantage to Amygdala-vmPFC RSFC

Our findings are supported by the rich literature on associations among neighborhood disadvantage and stressful life experiences and stress physiology, which in turn affect amygdala function. We showed that neighborhood disadvantage affects amygdala connectivity and propose that stressful experiences inherent to disadvantaged communities may mediate these effects. Despite the considerable heterogeneity across disadvantaged communities, findings suggest that such communities tend to have reduced social cohesion, lower accessibility to healthy food options and higher rates of food insecurity, higher rates of violent crime, lower educational attainment, and increased exposure to environmental neurotoxicants (Duncan 1996; Macintyre et al. 2002; Wilson et al. 2004; Braveman et al. 2005; Xue et al. 2005; Kohen et al. 2008; Meyer et al. 2014; Burt et al. 2016; Chang et al. 2016; DeVylder et al. 2018). The developing brain may be particularly vulnerable to the effects of this onslaught of neighborhood stressors by several mechanisms at different time points including prenatal transplacental stress hormone, inflammatory, and neurotoxicant insult and postnatal hypothalamic-pituitary-adrenal (HPA) axis dysregulation following early life stress. Indeed, children from more disadvantaged neighborhoods have higher basal cortisol levels (Finegood et al. 2017; Gianaros et al. 2017; Roubinov et al. 2018) and more cortisol dysregulation following a laboratory stressor (Hackman et al. 2012; Theall et al. 2017).

**Table 3:** Effects of age, neighborhood socioeconomic measures, and their interaction on amygdala-vmPFC connectivity

*N* = 127	Age (at mean neighborhood SES)		Age × % of neighbors < 150% poverty threshold		% of neighbors < 150% poverty threshold	
	*β*	*P*	*β*	*P*	*Β*	*P*
RBLA	−0.17	0.088	0.27	0.003^*^	0.002	0.98
LBLA	0.005	0.96	0.06	0.54	−0.05	0.57
RCMA	−0.12	0.27	0.16	0.10	0.01	0.94
LCMA	−0.05	0.63	.22	0.019	0.01	0.90
*N* = 127	Age (at mean neighborhood SES)		Age × % of neighbors ≥ age 25 with a HS Diploma		% of Neighbors ≥ age 25 with a HS Diploma	
	*β*	*P*	*β*	*P*	*Β*	*P*
RBLA	−0.17	0.089	−0.24	0.023	0.07	0.41
LBLA	0.002	0.98	−0.09	0.36	0.13	0.15
RCMA	−0.12	0.26	−0.17	0.10	0.09	0.33
LCMA	−0.05	0.65	−0.24	0.025	−0.003	0.97

*Notes:*

1. All models control for sex, head motion, pulse sequence, and ADHD diagnosis; reported betas are standardized; ^*^P < 0.05 with FDR-correction across 12 regression coefficient significance tests per neighborhood socioeconomic measure; SES = socioeconomic status; R/LBLA = right/left basolateral amygdala; R/LCMA = right/left centromedial amygdala.

Cortisol mediates some effects of stress on brain development, particularly in the amygdala. For example, maternal prenatal cortisol predicts amygdala functional connectivity in neonates (Graham et al. 2019), and both prenatal maternal cortisol (Buss et al. 2012) and postnatal child cortisol (Pagliaccio et al. 2014) predict amygdala structure. Furthermore, the amygdala and its connectivity with the ventromedial prefrontal cortex contribute to HPA axis function (Urry et al. 2006; Vaisvaser et al. 2013; Hakamata et al. 2017). Some studies have begun to investigate the influences of neighborhood disadvantage on inflammatory processes (Broyles et al. 2012; Smith et al. 2017), and it is known that environmental toxicants are distributed in a socioeconomically stratified manner (Cureton 2011; Vivier et al. 2011; Young et al. 2012; Morelli et al. 2017). Furthermore, there is evidence that inflammatory processes affect amygdala connectivity (Harrison et al. 2009; Graham et al. 2018; Mehta et al. 2018). These previous findings provide multiple biologically plausible pathways to explain our findings that neighborhood disadvantage has a measurable impact on child amygdala functional connectivity.

**Table 4:** The effects of age, neighborhood SES, and their interaction on basolateral amygdala-vmPFC functional connectivity, controlling for the interaction between household SES and age

*N* = 95		Neighborhood SES × age		Neighborhood SES		Household SES × age				Household SES			
						Middle SES > high SES		Low SES > high SES		Middle SES > high SES		Low SES > high SES	
		*β*	*P*	*β*	*P*	*β*	*P*	*β*	*P*	*β*	*P*	*β*	*P*
Parental education	RBLA	−0.70	0.001^*^	0.48	0.048	−0.74	0.001^*^	−0.32	0.20	−0.04	0.70	0.06	0.60
	LBLA	0.20	0.049	0.20	0.42	−0.62	0.009^*^	−0.62	0.020^*^	−0.03	0.81	−0.02	0.90
Hollingshead	RBLA	0.69	0.001^*^	0.53	0.026^*^	−0.73	0.002^*^	−0.37	0.14	0.04	0.68	−0.02	0.86
	LBLA	0.43	0.050	0.21	0.40	−0.63	0.008^*^	−0.59	0.023^*^	−0.01	0.93	0.03	0.78

*Notes:*

1. All models control for sex, head motion, pulse sequence, and ADHD diagnosis; reported betas are standardized; ^*^P < 0.05 with FDR-correction across 8 regression coefficient significance tests per household socioeconomic measure; SES = socioeconomic status; R/LBLA = right/left basolateral amygdala

### Precocious Development and the Stress Acceleration Hypothesis

Our study provides evidence supporting the “stress acceleration hypothesis” that stressful early life experiences promote precocious brain development in children in order to better cope with adverse environments (Callaghan and Tottenham 2016). Notably, findings suggest that the maturation of amygdala-vmPFC functional connectivity is characterized by shifts from positive to negative connectivity during an emotional face task (Gee, Humphreys, et al. 2013b) and near zero at rest (Jalbrzikowski et al. 2017). In our study, we show that children from less advantaged neighborhoods had, on average, near-zero amygdala-vmPFC RSFC and a greater likelihood of having negative connectivity than advantaged children, perhaps indicating earlier maturation. Importantly, this finding is in line with a previous study showing reduced amygdala-vmPFC connectivity during an emotional faces task in children who experienced early life caregiver adversity relative to typically developing children (Gee, Gabard-Durnam, et al. 2013a). Furthermore, we provide preliminary evidence that the effects of neighborhood characteristics on connectivity protect against anxiety symptoms. Such negative connectivity may confer resilience against symptoms in disadvantaged contexts, whereas positive connectivity may be protective in advantaged contexts. Importantly, although context-specific brain development following socioeconomic disadvantage appeared to confer benefits in the short term in our study, evidence also suggests that socioeconomic disadvantage does increase risk for anxiety over time (Ruscio et al. 2017). Similar to the disadvantaged participants in our study, anxiety and amygdala-vmPFC connectivity are inversely associated in adults (Etkin et al. 2009; Hahn et al. 2011; Kim et al. 2011). Longitudinal studies are required to understand the factors and developmental sequelae that potentially render these adaptations against anxiety insufficient in the long term. We note that these behavioral results are preliminary given reduced sample size and power. Nevertheless, in addition to supporting the stress acceleration hypothesis, they suggest that neighborhood SES may explain the inconsistent direction in associations between amygdala-vmPFC RSFC and anxiety symptoms in children (Gee, Gabard-Durnam, et al. 2013a; Hamm et al. 2014; Qin et al. 2014; Wolf and Herringa 2016).

### Neighborhood and Household SES

Previous work has shown the profound effects of neighborhood characteristics on children’s behavior, particularly in experimental designs that control for household SES (Brooks-Gunn et al. 1993; Chase-Lansdale and Gordon 1996; Ludwig et al. 2001; Leventhal and Brooks-Gunn 2003; Jackson et al. 2009; Leventhal and Dupere 2011). Our brain findings add to emerging literature on the effects of neighborhood SES on neural development (Tooley et al. 2019; Ramphal et al. 2020) that may be dissociable from household effects (Gianaros et al. 2017; Marshall et al. 2018; Miller et al. 2018; Tomlinson et al. 2020). Indeed, although household and neighborhood disadvantage are often correlated (*r* = 0.36–0.37 in our study), the aforementioned domains of disadvantage that may contribute to neighborhood-brain associations (e.g., neighborhood social cohesion, neighborhood violence, environmental neurotoxicant burden, and food accessibility) are spatially distributed. That is, since they operate at a neighborhood level, measuring them at the neighborhood level offers the best operationalization of how these domains are distributed (Garner and Raudenbush 1991; Sampson et al. 1997; Browning and Cagney 2003; Brulle and Pellow 2006; Walker et al. 2010). Thus, neighborhood disadvantage likely captures the nuances of their distribution more precisely than household disadvantage, which can vary widely within a single neighborhood. For example, a highly educated individual can live in a polluted neighborhood. A poor family can live in a cohesive neighborhood (Farah 2017). As such, neighborhood and household processes are meaningfully distinguishable and future studies of SES should continue to treat them as distinct, albeit related measures.

Although other studies have identified associations between household SES and brain structure, function, and connectivity (Hackman and Farah 2009), we did not. We analyzed household disadvantage in two ways: first, by parental education and second, by Hollingshead score, a measure of household SES that includes consideration of both occupation and education. A more complete examination of household SES would include a measure of household income, which we did not have because our sample consisted of pooled data from several case-control neuroimaging studies. One study examining amygdala-vmPFC connectivity at a single age found that lower income was associated with more negative connectivity (Hanson et al. 2019). Our failure to detect an association between household SES and functional connectivity could be due to the absence of strictly economic household characteristics in our study. Notably this study did not include a measure of neighborhood SES*.* Another possibility is that neighborhood and household SES interact to contribute to brain development and behavior. Additional work designed to test these interactive effects is necessary to understand the unique and shared contributions of various scales of SES to child brain and emotional development.

### What is Normative?

Prior studies have posited that amygdala-vmPFC connectivity normally shifts from positive to negative or near-zero with increasing age, both with cross-sectional (Gee, Humphreys, et al. 2013b) and longitudinal (Jalbrzikowski et al. 2017) designs. Although the cross-sectional nature of our data limits our ability to interpret our findings as strictly developmental, we provide evidence that this protracted reduction in connectivity with increasing age may be limited to participants residing in highly advantaged communities characterized by low poverty rates. Interestingly, age was unassociated with connectivity among the middle SES group which had similar high school graduation and poverty rates as the high SES group. This could be because these specific components of the ADI may not represent the neighborhood characteristics that best distinguish highly advantaged neighborhoods. For example, neighborhoods categorized in our sample as being high, middle, and low SES group had bachelor’s degree attainment rates of 56%, 39%, and 15%, respectively. Highly advantaged communities are also characterized by consistent improvement in the domains of safety, public space, and school quality (Solari 2012), which might further differentiate the effects of high and middle SES neighborhoods.

**Table 5:** Effects of the interaction between age, neighborhood socioeconomic status, and basolateral amygdala-vmPFC resting-state functional connectivity on the Anxious/Depressed subscale of the CBCL.

N = 65	RBLA-vmPFC RSFC × Neighborhood SES × Age		LBLA-vmPFC RSFC × Neighborhood SES × Age	
	Β	*P*	β	*P*
Area Deprivation Index	−3.29	.007	−1.53	.24
% of Neighbors < 150% Poverty Threshold	−2.85	.028	−2.13	.11
% of Neighbors ≥ age 25 with a HS Diploma	2.96	.035	1.43	.39

*Notes:*

1. All models control for sex, head motion, pulse sequence, and ADHD diagnosis; reported betas are standardized. SES = socioeconomic status; R/LBLA = right/left basolateral amygdala; RSFC = resting-state functional connectivity.

Among individuals from less advantaged neighborhoods, the absence of age effects and the presence of near-zero connectivity suggest that this pattern of mature connectivity developed prior to the youngest age in our sample. Our findings are consistent with previous work demonstrating that age-brain associations can be strongly biased by sample demographic composition (LeWinn et al. 2017). Altogether, these findings underscore the necessity of examining and reporting the effects of neighborhood socioeconomic status in studies of brain development (Farah 2017).

Our findings add to an emerging literature documenting that socioeconomic deprivation affects the developing brain (Hackman and Farah 2009; Farah 2018). Though our findings point to a mechanism of short-term resilience, other social factors and biological mechanisms likely contribute to known associations between disadvantage and mental illness and additional work should investigate these (Aneshensel and Sucoff 1996; Ross 2000; Browning and Cagney 2003; Luby et al. 2013). Furthermore, our study should not dissuade continued efforts to improve the environments in which children develop. Both neighborhood- (Ludwig et al. 2012; South et al. 2018) and household-level (Costello et al. 2003) socioeconomic interventions have successfully improved mental health. Future studies should also further parse the effects of neighborhood- and household-level intervention to better understand the most efficacious ways of improving mental health disparities.

## Supplementary Material

ses_amyg_vmpfc_supplement_final_tgaa033
