# Supplementary material for: Associations between Amygdala-Prefrontal Functional Connectivity and Age Depend on Neighborhood Socioeconomic Status
Source: Cereb Cortex Commun. 2020 Jul 23;1(1):tgaa033. doi: 10.1093/texcom/tgaa033 (PMC7503474; doi:10.1093/texcom/tgaa033)
Supplement: ses_amyg_vmpfc_supplement_final_tgaa033 [file ses_amyg_vmpfc_supplement_final_tgaa033.docx]

Amygdala-Prefrontal Functional Connectivity Depends on Neighborhood Socioeconomic Status

SUPPLEMENTARY METHODS

Household SES Measures

Hollingshead occupation scores range from 1 to 9. Examples for occupation scores include: 1=babysitter, janitor; 2=sanitation worker, waiter; 3=health aide, housekeeper; 4=carpenter, electrician; 5=health trainee, dental assistant; 6=photographer, sale manager; 7=social worker, teacher; 8=pharmacist, accountant; 9= physician, lawyer (Hollingshead 1975).

Our ordinal educational attainment variable was coded according to the following scores ranging 1 to 7: 1=less than middle school; 2=completed middle school; 3=some high school; 4=completed high school/attained GED; 5=some college/associate’s degree; 6=bachelor’s degree; 7=graduate degree.

Main Analyses

*Hypothesis 1.* Age, neighborhood SES, and RSFC; n=127

Sample R code: lm(data, LBLA_vmPFC_RSFC ~ sex + ADHD + pulse_sequence + motion + **age*ADI_lowSES** + **age*ADI_middleSES**)

*Hypothesis 2.* Age, neighborhood SES, household SES, and RSFC; n=95

lm(data, LBLA_vmPFC_RSFC ~ sex + ADHD + pulse_sequence + motion + **age*ADI_lowSES** + **age*ADI_middleSES** + **age*Hollingshead**)

*Hypothesis 3.* Age, neighborhood SES, RSFC, and anxiety; n=65

lm(data, CBCL_anxiousdepressed ~ sex + ADHD + pulse_sequence + motion + **LBLA_vmPFC_RSFC*age*ADI**)

Post-hoc Analyses

*Global signal regression.* We performed our analysis for *Hypothesis 1* using an identical preprocessing pipeline that included global signal regression.

*Quadratic age effects.* Given previous findings of quadratic age effects on brain development, we performed our analysis for *Hypothesis 1* with additional terms representing the interaction between age-squared and neighborhood SES.

*Influence of ADHD participants.* Multiple methods were employed to address the possibility that ADHD diagnosis might have driven any observed neighborhood effects. First, we included ADHD diagnosis as a covariate in all analyses. Second, we tested whether the three-way interaction between age, neighborhood SES, and ADHD diagnosis predicted connectivity.

lm(data, LBLA_vmPFC_RSFC ~ sex + pulse_sequence + motion + **ADHD*age*ADI_lowSES** + **ADHD*age*ADI_middleSES**

Third, we tested the two-way interaction between ADHD and age.

lm(data, LBLA_vmPFC_RSFC ~ sex + pulse_sequence + motion + age + ADI_lowSES + ADI_middleSES + **ADHD*age**)

SUPPLEMENTARY RESULTS

Neighborhood SES

Neighborhood SES groups did not differ in composition with respect to sex (chi-square=2.59, p=.27), age (F=0.59, p=.56), or ADHD diagnosis (chi-square=4.23, p=.12). Consistent with epidemiological data, children with ADHD were more disadvantaged than healthy children in terms of ADI score (mean difference= 16.6, p=.02). Nevertheless, ADHD diagnosis was thoroughly ruled out as a confound in all analyses as described in Supplementary Methods: Post-Hoc Analyses. The proportion of neighbors under 150% of the poverty line and the proportion of individuals 25 years old or older with a high school diploma were associated with one another (r=-.72, p<.001) and also associated with ADI (r=.63, p<.001 and r=-0.63, p<.001, respectively). Distributions of all socioeconomic measures are presented in Figure S1. Of the 127 participants with useable MRI and neighborhood SES data, 95 had complete household SES data.

Behavioral Subsample

65 subjects (ages 5-17) had complete CBCL Anxious/Depressed and neighborhood SES data and were included in behavioral analysis (8 from learning study, 13 from ADHD study #1, 34 from ADHD study #2, 0 from the bulimia nervosa study, and 10 from the substance use study). Relative to our main sample with 127 subjects, this behavioral subsample had a greater proportion of male (52% vs. 74%, χ=7.6, p=.006) and ADHD participants (43% vs. 65% χ^2^=7.5, p=.006). 24 participants were from high SES neighborhoods, 21 were from middle, and 20 were from low SES neighborhoods.

Anxiety

There were no associations between CBCL Anxiety/Depression T-score and neighborhood SES, household SES, or sex (ps>.05). Youth with ADHD had more anxiety/depression symptoms than those without ADHD mean difference=5.00, p=.001); as in other analyses, we controlled for ADHD in brain-behavior analyses. This analysis Did

All of the following models control for sex, head motion, pulse sequence, and ADHD diagnosis; reported betas are standardized. SES=socioeconomic status; L/RBLA=left/right basolateral amygdala; L/RCMA=left/right centromedial amygdala


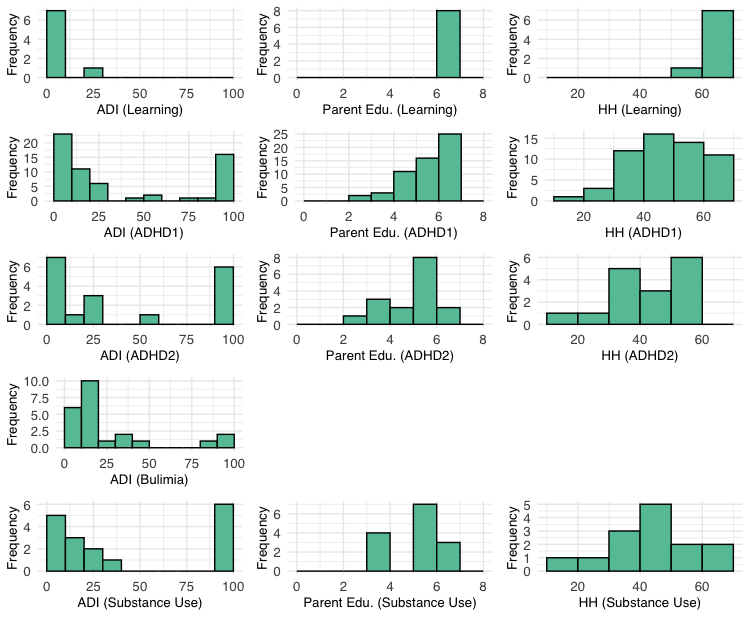


Figure S1. Neighborhood and household SES in all participants by study. The bulimia study did not collect household SES.


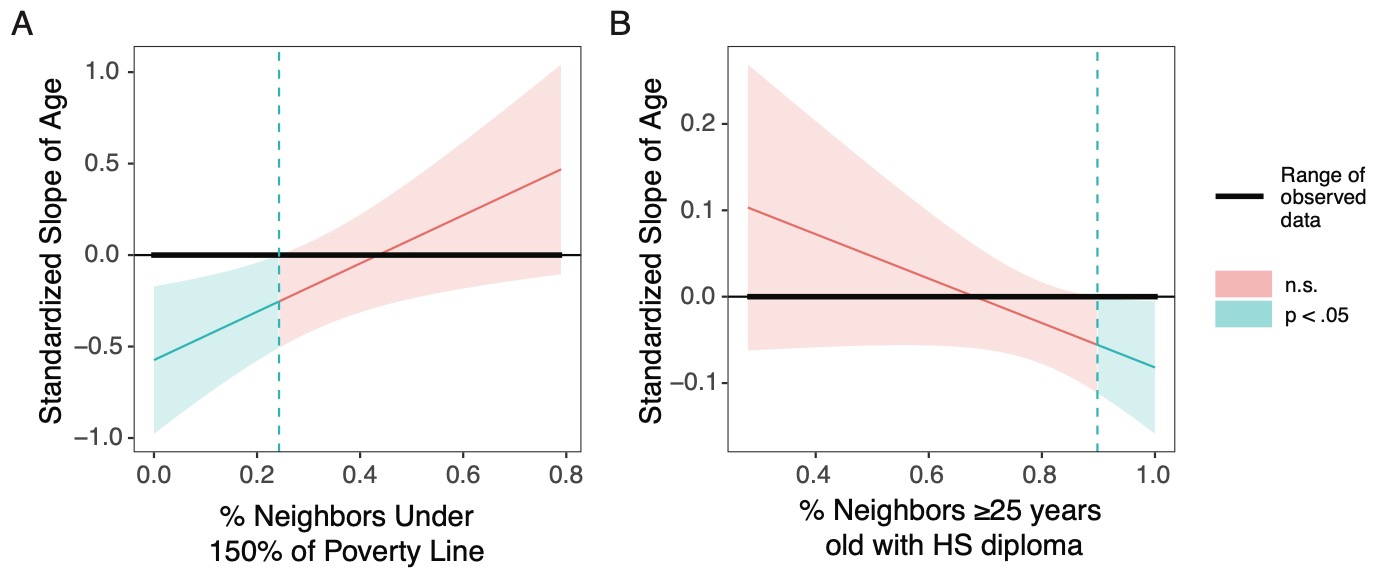


Figure S2. Johnson-Neyman plots for the effects of neighborhood poverty and educational attainment on associations between age and RBLA-vmPFC connectivity.

Table S1. Sample characteristics and MRI acquisition parameters in each original study. There were three distinct pulse sequences which were coded as Learning, ADHD1, or All Others.

|  | Study | | | | |
| --- | --- | --- | --- | --- | --- |
|  | Learning | ADHD1 | ADHD2 | Bulimia | Substance Use |
| Sample Characteristics | | | | | |
| n | 8 | 61 | 18 | 23 | 17 |
| n with ADHD | 0 | 33 | 16 | 0 | 5 |
| Age range (years) | 7-10 | 6-25 | 5-25 | 12-19 | 13-22 |
| Recruitment strategies | Schools | Flyers in local community  Internet and newspaper ads | | | |
|  | Private practices | |  |  |  |
| MRI Acquisition Parameters | | | | | |
| TR (s) | 2 | 2 | 2.2 | 2.2 | 2.2 |
| # of repetitions | 140 | 155 | 140 | 140 | 140 |
| TE (ms) | 30 | 30 | 30 | 30 | 30 |
| Flip Angle (°) | 77 | 90 | 90 | 90 | 90 |
| # of runs | 2 | 2 | 2 | 2 | 2 |
| Length of resting scan | 9 min, 20 sec | 10 min, 20 sec | 10 min, 16 sec | 10 min, 16 sec | 10 min, 16 sec |
| Pulse Sequence | Learning | ADHD1 | All Others | | |

Table S2. Neighborhood characteristics of SES groups in our sample.

|  | Neighborhood SES Group | | |
| --- | --- | --- | --- |
|  | High n=48 | Middle n=48 | Low n=31 |
| % of Individuals <150% Poverty Threshold | 20.0 | 25.9 | 54.3 |
| % of Individuals ≥ age 25 with a HS Diploma | 89.4 | 82.9 | 60.1 |

Table S3. Effects of age, neighborhood SES, and their interaction on amygdala-vmPFC connectivity with global signal regression.

| N=127 | Age (in high neighborhood SES participants) | | Age × Neighborhood SES | | | | Neighborhood SES | | | |
| --- | --- | --- | --- | --- | --- | --- | --- | --- | --- | --- |
|  |  |  | Middle SES> High SES | | Low SES > High SES | | Middle SES> High SES | | Low SES > High SES | |
|  | β | p | β | p | β | p | β | p | β | p |
| LBLA | -0.20 | .15 | 0.41 | .039 | 0.20 | .38 | -0.51 | .01 | -0.71 | .002 |
| RBLA | -0.54 | .14 | 0.59 | .003 | 0.58 | .01 | -0.60 | .003 | -0.47 | .038 |
| LCMA | -0.20 | .18 | 0.09 | .67 | 0.41 | .099 | -0.32 | .14 | -0.30 | .22 |
| RCMA | -0.35 | .020 | 0.27 | .20 | 0.49 | .043 | -0.46 | .029 | -0.37 | .14 |

Table S4. Effects of age, neighborhood SES analyzed continuously, and their interaction on amygdala-vmPFC connectivity.

| N=127 | Age (at mean neighborhood SES) | | Age × Neighborhood SES | | Neighborhood SES | |
| --- | --- | --- | --- | --- | --- | --- |
|  | β | p | β | p | β | p |
| LBLA | -0.01 | .89 | 0.03 | .72 | -0.22 | .015 |
| RBLA | -0.18 | .07 | 0.18 | .058 | -0.09 | .34 |
| LCMA | -0.06 | .54 | 0.13 | .16 | -0.12 | .21 |
| RCMA | -0.12 | .26 | 0.10 | .29 | -0.02 | .80 |

Table S5. Effects of the interaction between age^2^ and neighborhood SES on BLA -vmPFC connectivity.

| N=127 | Age^2^ (in high neighborhood SES participants) | | Age^2^ × Neighborhood SES | | | |
| --- | --- | --- | --- | --- | --- | --- |
|  |  |  | Age^2^ ×Middle Neighborhood SES> High SES | | Age^2^ ×Low Neighborhood SES > High SES | |
|  | β | p | β | p | β | p |
| LBLA | 0.14 | .34 | 0.10 | .63 | 0.01 | .97 |
| RBLA | 0.06 | .67 | 0.26 | .20 | 0.49 | .043 |

Table S6. Effects of the interaction between age, neighborhood SES, and ADHD diagnosis on BLA -vmPFC connectivity.

| N=127 | Age × Middle Neighborhood SES> High SES × HC> ADHD | | Age × Low Neighborhood SES > High SES × HC> ADHD | | Middle Neighborhood SES> High SES × HC> ADHD | | Low Neighborhood SES > High SES × HC> ADHD | |
| --- | --- | --- | --- | --- | --- | --- | --- | --- |
|  | β | p | β | p | β | p | β | p |
| LBLA | -0.61 | .18 | -0.15 | .78 | 0.47 | .31 | 0.17 | .73 |
| RBLA | -0.12 | .79 | 0.08 | .88 | -0.22 | .64 | -0.42 | .40 |

Table S7. Effects of the interaction between age and ADHD diagnosis on BLA-vmPFC connectivity.

| N=127 | Age × ADHD | | ADHD | |
| --- | --- | --- | --- | --- |
|  | β | p | β | p |
| LBLA | 0.22 | .30 | -0.22 | .28 |
| RBLA | 0.25 | .25 | -0.01 | .96 |

Table S8. Effects of the interaction between neighborhood SES and age on BLA-vmPFC functional connectivity, controlling for the interaction between Hollingshead (alternatively determined by maximal household occupation/education) and age

| N=95 | | Age × Neighborhood SES | | | | Neighborhood SES | | | | Household SES × Age | | Household SES | |
| --- | --- | --- | --- | --- | --- | --- | --- | --- | --- | --- | --- | --- | --- |
|  |  | Middle SES> High SES | | Low SES > High SES | | Middle SES> High SES | | Low SES > High SES | |  |  |  |  |
|  |  | β | p | β | p | β | p | β | p | β | p | β | p |
| Hollingshead (maximal household occupation/educated) | RBLA | 0.68 | .001 | 0.52 | .026 | -0.72 | .002 | -0.38 | .12 | 0.02 | .83 | -0.07 | .53 |
|  | LBLA | 0.43 | .047 | 0.20 | .40 | -0.62 | .010 | -0.61 | .017 | -0.05 | .67 | -0.04 | .76 |

Table S9. Effects of the interaction between neighborhood SES and age on BLA-vmPFC functional connectivity, controlling for the interaction between age and either Hollingshead groups or parental education groups. Hollingshead groups were defined based on the following ranges: 8-39 (unskilled laborers, semiskilled workers, skilled craftsmen, n=24), 40-54 (minor professionals, medium business, n=42), 55-66 (major business, professional, n=29), Hollingshead, 1975. Parental education groups were defined according to high school graduation(n=13), some college attendance/bachelor’s degree attainment(n=44), or graduate degree attainment (n=38).

| N=95 | | Age × Neighborhood SES | | | | Neighborhood SES | | | | Household SES × Age | | | | Household SES | | | |
| --- | --- | --- | --- | --- | --- | --- | --- | --- | --- | --- | --- | --- | --- | --- | --- | --- | --- |
|  |  | Middle SES> High SES | | Low SES > High SES | | Middle SES> High SES | | Low SES > High SES | | Middle SES> High SES | | Low SES > High SES | | Middle SES> High SES | | Low SES > High SES | |
|  |  | β | p | β | p | β | p | β | p | β | p | β | p | β | p | β | p |
| Hollingshead Groups | RBLA | 0.68 | .002 | .52 | .03 | -0.73 | .003 | -0.37 | .14 | 0.01 | .98 | -0.05 | .85 | 0.03 | .91 | 0.12 | .70 |
|  | LBLA | 0.95 | .076 | .24 | .33 | -0.63 | .012 | -0.61 | .02 | 0.15 | .53 | -0.11 | .71 | -0.001 | .99 | 0.05 | .87 |
| Parental Education Groups | RBLA | 0.70 | .001 | .51 | .03 | -0.76 | .001 | -0.31 | .21 | 0.10 | .63 | 0.07 | .84 | -0.07 | .75 | -0.32 | .34 |
|  | LBLA | 0.44 | .051 | 0.44 | .42 | -0.63 | .009 | -0.59 | .02 | 0.04 | .87 | 0.10 | .79 | -0.07 | .78 | -0.09 | .81 |

Table S10. Effects of the interaction between neighborhood poverty and age on BLA-vmPFC functional connectivity, controlling for the interaction between household SES and age.

| N=95 | | Age × % of Neighbors <150% Poverty Threshold | | % of Neighbors <150% Poverty Threshold | | Age × Household SES | | Household SES | |
| --- | --- | --- | --- | --- | --- | --- | --- | --- | --- |
|  |  | β | p | β | p | β | p | β | p |
| Parental Education | LBLA | 0.26 | .63 | -0.14 | .81 | 0.02 | .85 | -0.01 | .96 |
|  | RBLA | 1.58 | .003 | 0.58 | .32 | 0.16 | .18 | 0.10 | .44 |
| Hollingshead | LBLA | 0.21 | .68 | -0.03 | .96 | 0.0001 | .99 | 0.05 | .69 |
|  | RBLA | 1.44 | .003 | 0.41 | .45 | 0.14 | .23 | 0.03 | .84 |

Table S11. Effects of the interaction between household SES and age on BLA-vmPFC functional

connectivity.

| N=95 | | Age | | Age × Household SES | | Household SES | |
| --- | --- | --- | --- | --- | --- | --- | --- |
|  |  | β | p | β | p | β | p |
| Parental Education | LBLA | -0.02 | .84 | -0.004 | .97 | 0.01 | .93 |
|  | RBLA | -0.17 | .16 | -0.02 | .83 | 0.02 | .84 |
| Hollingshead | LBLA | -0.03 | .79 | -0.02 | .89 | 0.05 | .65 |
|  | RBLA | -0.15 | .19 | 0.02 | .87 | -0.03 | .77 |

Table S12. Effects of the interaction between age, household SES, and BLA-vmPFC resting-state functional connectivity on the Anxious/Depressed subscale of the CBCL

| N=61 | Age × Household SES × LBLA | | Age × Household SES × RBLA | |
| --- | --- | --- | --- | --- |
|  | β | p | β | p |
| Parental Education | 1.88 | .21 | 1.16 | .41 |
| Hollingshead | 3.05 | .08 | 2.26 | .09 |

References

Hollingshead AB. 1975. Four-factor index of social status. New Haven, CT: Yale University Press.
